# A highly potent compound W-1-32 targeting XIAP protein inhibits proliferation and metastasis of esophageal squamous cell carcinoma

**DOI:** 10.3389/fphar.2026.1902572

**Published:** 2026-08-19

**Authors:** Yuxiang Jin, Zifen Ma, Qianyu Han, Qian Yuan, Siang Zhang, Qiang Lyu, Mingwei Jiang, Weiheng Xu, Lei Xue

**Affiliations:** 1 Department of Thoracic Surgery, Second Affiliated Hospital of Naval Medical University, Shanghai, China; 2 School of Pharmacy, Second Military Medical University, Shanghai, China

**Keywords:** cell proliferation and migration, computational analysis, esophageal cancer cells, W-1-32, XIAP

## Abstract

**Background:**

China bears the highest global burden of esophageal cancer, with esophageal squamous cell carcinoma representing the predominant histological subtype. The poor clinical outcome of ESCC is largely attributable to its aggressive invasiveness and early metastatic spread. Currently, effective first-line targeted therapies remain limited, underscoring the need to identify actionable molecular targets and develop novel targeted agents for ESCC.

**Methods:**

XIAP expression was examined by Western blot analysis. Cell proliferation was assessed using CCK-8 assays, while cell migration and invasion were evaluated by wound-healing and Transwell assays. The interaction between W-1-32 and XIAP was investigated using drug affinity responsive target stability and cellular thermal shift assays. Molecular docking and molecular dynamics simulations were employed to explore the binding mode of W-1-32 to XIAP.

**Results:**

A small-molecule compound, W-1-32, was identified as a modulator of XIAP. Computational analyses suggested a stable binding mode between W-1-32 and XIAP mediated by hydrogen bonding and noncovalent interactions, which was further supported by DARTS and CETSA assays. Treatment with W-1-32 reduced XIAP protein abundance in ESCC cells and was associated with decreased cell proliferation and migratory capacity. Notably, W-1-32 exhibited lower cytotoxicity toward normal esophageal epithelial cells compared with ESCC cells, indicating a degree of tumor selectivity *in vitro*.

**Conclusion:**

Our results confirmed that W-1-32 significantly reduces ESCC cell proliferation and migration, which may be attributed to its binding to XIAP and the consequent reduction of XIAP protein levels. These findings identify W-1-32 as a XIAP-modulating compound with selective *in vitro* activity against ESCC cells, warranting further *in vivo* pharmacokinetic, efficacy, and safety evaluation before translational consideration.

## 1 Introduction

Esophageal cancer is a highly aggressive malignancy of the digestive tract with a substantial global disease burden. In 2020, approximately 604,000 new cases and 544,000 deaths were reported worldwide, ranking seventh in incidence and sixth in mortality among all cancers (Huang et al., 2021). China accounts for the highest prevalence of esophageal cancer, where more than 90% of cases are diagnosed as esophageal squamous cell carcinoma (ESCC) (Yang and Chen, 2021). Owing to its early metastatic propensity, resistance to conventional chemotherapy, and lack of effective targeted therapies, the 5-year overall survival rate of ESCC remains dismal at approximately 15–20 (Reichenbach et al., 2019; Yang et al., 2020). These clinical challenges highlight an urgent need to identify novel molecular targets and develop effective targeted therapeutic agents for ESCC.

X-linked inhibitor of apoptosis protein (XIAP) is a 473 amino acid protein with three BIR domains, one RING domain, and one UBA domain, encoded by genes mapped to the Xq25 region of the X chromosome (Muñoz et al., 2019). XIAP specifically inhibits caspases-3, -7, and -9 through its BIR domains, directly suppressing caspase enzyme activity (Muñoz et al., 2019; Cossu et al., 2019). It is highly expressed in various cancers and is associated with disease progression and poor prognosis (Lou et al., 2021; Zohny et al., 2019; Łupicka-Słowik et al., 2020). Previous studies have demonstrated that XIAP promotes ESCC invasion and metastasis (Jin et al., 2019), making it a promising therapeutic target.

Natural products represent a rich and structurally diverse source of bioactive molecules, offering unique chemical scaffolds that frequently exhibit favorable biological compatibility and multi-target potential (Mishra et al., 2014). Beyond conventional bioactivity screening, modern drug discovery increasingly integrates quantum chemical calculations to elucidate the intrinsic electronic properties underlying ligand–target recognition and reactivity (Ginex et al., 2024; Galvani et al., 2022). Density functional theory (DFT)–based analyses, including frontier molecular orbital distributions, molecular electrostatic potential (MEP) mapping, and conceptual DFT descriptors, provide valuable insights into molecular stability, electrophilic–nucleophilic behavior, and noncovalent interaction propensity, which are closely related to pharmacological activity and binding selectivity (Guan et al., 2025; Uzun et al., 2019).

In the present study, we identified W-1-32, a novel natural compound, as a selective inhibitor of XIAP. W-1-32 effectively suppresses ESCC cell proliferation and migration while downregulating XIAP expression. To further elucidate the pharmacological features of W-1-32 at the molecular level, we employed DFT-based quantum chemical calculations to characterize its electronic structure, chemical reactivity, and potential interaction sites. The molecular dynamics simulation and metadynamics simulation clearly revealed the binding models between XIAP and W-1-32. These computational analyses were integrated with molecular docking and biological assays to inform a structure–activity relationship linking the intrinsic electronic properties of W-1-32 with its XIAP-targeted antitumor effects. Collectively, our findings suggested that W-1-32 represents a promising XIAP-targeting lead compound warranting further preclinical evaluation in ESCC and highlighted the utility of quantum chemical approaches in natural product–based anticancer drug development.

## 2 Materials and methods

### 2.1 Bioinformatics analysis

#### 2.1.1 Pan-cancer analysis

Gene expression data and corresponding clinical information for pan-cancer analysis were obtained from The Cancer Genome Atlas (TCGA) database (https://portal.gdc.cancer.gov/). Transcriptome data for esophageal squamous cell carcinoma (ESCC) and adjacent normal tissues were downloaded from TCGA and the Gene Expression Omnibus (GEO) database (https://www.ncbi.nlm.nih.gov/geo/), including the GSE23400, GSE53625, and GSE38129 datasets. Single-cell RNA sequencing data for ESCC were obtained from the GEO dataset GSE196756. Genetic alteration analysis of XIAP was performed using the cBioPortal for Cancer Genomics platform (https://www.cbioportal.org/), which provided a summary of mutation frequencies and alteration types across different cancer types. The TIMER2.0 database (https://compbio.cn/timer2/) was used to evaluate the correlation between XIAP expression and immune cell infiltration. Spearman’s rank correlation coefficient was calculated to assess the relationship between XIAP expression levels and the abundance of various immune cell types. Overall survival analysis was performed using the TCGA ESCC cohort, Kaplan–Meier survival curves were generated, and differences between groups were evaluated using the log-rank test (Yang et al., 2025).

#### 2.1.2 Single-cell sequencing analysis

Single-cell RNA sequencing data from the GSE196756 dataset were processed using the Seurat package (version 4.3.0) in R software. Cells were filtered by excluding those with mitochondrial gene content >20% and retaining cells with nFeature_RNA between 200 and 5,000. Data normalization, scaling, and principal component analysis (PCA) were performed. Dimensionality reduction was conducted using Uniform Manifold Approximation and Projection (UMAP). Cell clusters were identified using the FindClusters function with a resolution of 0.1. Marker genes for each cluster were identified using the FindAllMarkers function, and cell types were annotated based on canonical marker genes. The expression distribution of XIAP across different cell clusters was visualized using the FeaturePlot and BubblePlot functions (Gao et al., 2025).

#### 2.1.3 Differential expression analysis and functional enrichment analysis

To investigate the biological processes associated with XIAP overexpression in ESCC, patients from the TCGA ESCC cohort were divided into high- and low-expression groups based on the median XIAP expression level. Differential expression analysis was performed using the limma package (version 3.56.2) in R software. Genes with an adjusted P-value <0.05 and |log2fold change| > 0.5 were considered differentially expressed genes (DEGs). Volcano plots were generated using the ggplot2 package (version 3.5.0). Gene Ontology (GO) enrichment analysis and Kyoto Encyclopedia of Genes and Genomes (KEGG) pathway enrichment analysis were performed using the clusterProfiler package (version 4.4.0) in R. Gene Set Enrichment Analysis (GSEA) was conducted using the fgsea package (version 1.36.2), referencing the Hallmark gene sets from MSigDB (version 7.5.1) to identify gene sets significantly enriched in the XIAP-high expression group compared with the XIAP-low group (Wa et al., 2024).

### 2.2 Chemicals and reagents

W-1-32 (purity > 99%, MW: 794.4225) was dissolved in DMSO at a final concentration below 0.1% and prepared by the School of Pharmacy, Naval Medical University. Fetal bovine serum (FBS) was obtained from Kingmorn (Shanghai, China). RPMI-1640 medium, 100 U/mL penicillin, and 100 μg/mL streptomycin were procured from Yuanpei (China). The cell counting kit-8 (CCK-8) was obtained from Shanghai Taoshu Biotechnology Co., Ltd. Radioimmunoprecipitation assay (RIPA) lysis buffer was procured from Biyuntian (China). The XIAP antibody (Cat. 22228-1AP) was acquired from Proteintech (China), and the GAPDH antibody (Cat. 60004-1Ig) from Affinity Biosciences (USA). The ReverTra cDNA synthesis kit was obtained from Takara (Japan). The transwell chamber was sourced from Corning (New York, USA). Pronase solution (Cat. 10165921001) was acquired from Roche and M-PER lysate (Cat. 78501) from Thermo Fisher. All other chemicals and reagents were of analytical grade.

#### 2.2.1 Density functional theory calculations

Density functional theory (DFT) calculations were performed using the PySCF package. The HQY molecular structure was imported from a MOL2 file, with atomic coordinates extracted and the total charge assigned when available. Spin multiplicity was determined from the total electron count, assuming closed-shell configurations for even-electron systems. Calculations were carried out at the B3LYP/6-311+G(d,p) level with level-3 numerical integration grids and a SCF convergence threshold of $1\times 10^{-9}$ Hartree. HOMO and LUMO energies were obtained from the converged Kohn–Sham orbitals, and orbital isosurfaces were generated by exporting the corresponding molecular orbitals as Gaussian cube files (grid resolution: 0.25 Bohr) for visualization using VMD2 (version2) software (https://www.ks.uiuc.edu/Research/vmd/vmd2intro/).

Global reactivity descriptors, including chemical hardness (η), chemical softness (S), electronegativity (χ), chemical potential (μ), and electrophilicity index (ω), were calculated under Koopmans’ approximation. Chemical hardness was defined as $\eta =\left(E_{\text{LUMO}}-E_{\text{HOMO}}\right)/2$ , and chemical softness was calculated as $S=1/\left(2\eta \right)$ . Electronegativity was estimated as $\chi =-\left(E_{\text{HOMO}}+E_{\text{LUMO}}\right)/2$ , while the chemical potential was defined as μ=−χ . The electrophilicity index was calculated according to $\omega =\mu^{2}/\left(2\eta \right)$ . All quantities were reported in electronvolt (eV).

#### 2.2.2 Molecular electrostatic potential (MEP) analysis

Molecular electrostatic potential (MEP) maps were calculated using the PySCF package at the B3LYP/6-311+G(d,p) level of theory. The MEP was evaluated from the converged Kohn–Sham electron density obtained with level-3 numerical integration grids and a self-consistent field (SCF) energy convergence threshold of $1\times 10^{-9}$ Hartree. The electrostatic potential was computed on a three-dimensional Cartesian grid with dimensions of 80×80×80 points, extending 4.0 Bohr beyond the outermost atoms in each direction. Gaussian cube files were generated using the cubegen.mep module in PySCF for subsequent visualization.

Regions exhibiting negative electrostatic potential were assigned as potential electrophile-interaction and hydrogen-bond-accepting sites, whereas regions with positive electrostatic potential were considered indicative of nucleophile-interaction and hydrogen-bond-donating centers. The predicted reactive sites were further interpreted in combination with frontier molecular orbital (HOMO/LUMO) spatial distributions and global conceptual DFT descriptors, including chemical hardness, electronegativity, and electrophilicity index.

### 2.3 Cell culture

The human normal esophageal epithelial cell line Het-1A and human ESCC cell lines (ECa109, KYSE150) were obtained from the Shanghai Institute of Life Sciences Cell Bank Center (Shanghai, China). Het-1A cells were cultured in BEGM supplemented with 10% FBS and 1% penicillin-streptomycin. All human ESCC cell lines were maintained in RPMI-1640 medium with 10% FBS and 1% penicillin-streptomycin. Cells were incubated at 37 °C in a humidified atmosphere with 5% CO2.

### 2.4 Cell transfection

Recombinant lentiviral vectors were obtained from Obio Technology Co., Ltd., (Shanghai, China). Cells were infected with fresh lentiviral vectors carrying either the vector control (sh-Ctrl) or sh-XIAP construct in a culture medium for 24 h. Subsequently, cells were maintained in a puromycin-containing medium (2 μg/mL) for 72 h. XIAP mRNA and protein expression levels in sh-Ctrl and sh-XIAP constructs were analyzed using quantitative polymerase chain reaction and Western blot techniques.

### 2.5 Drug affinity responsive target stability (DARTS)

The principle of the target stability assay in drug affinity reactions is that when a small-molecule compound binds to a protein, this interaction stabilizes the target protein structure, rendering it resistant to protease digestion. This technique is applicable for identifying target proteins of any small-molecule compound. In this study, KYSE150 cells were cultured to 70%–80% confluency and then lysed with M-PER to extract soluble proteins. The cell lysate was incubated at room temperature with varying drug concentrations for 60 min on a shaker to facilitate binding. Each sample was then treated with either pronase solution (1:3,000) or buffer alone (undigested) and incubated for 30 min at room temperature. Protein digestion was halted, and the lysate was mixed with 4× protein buffer and heated at 100 °C for 10 min before Western blot analysis.

### 2.6 The cellular thermal shift assay (CETSA)

Eca109 and KYSE150 cells were cultured to 70%–80% confluency and incubated with either vehicle (DMSO) or W-1-32 (30 μM) for 6 h. After treatment, cell pellets were resuspended in ice-cold phosphate-buffered saline (PBS) containing protease and phosphatase inhibitors. The lysates were then divided into 50 μL aliquots, transferred to 0.2 mL polymerase chain reaction tubes, and heated at the specified temperature for 5 min. Samples were immediately placed on ice, subjected to three freeze-thaw cycles with liquid nitrogen, and centrifuged at 20,000 × *g* for 20 min at 4 °C to collect soluble proteins. XIAP expression was analyzed using the Western blot technique (Bio-Rad, Mini PROTEAN).

### 2.7 CCK-8 assay

The effect of W-1-32 on ESCC cell proliferation was evaluated using the CCK-8 assay. Eca109 and KYSE150 cells were seeded in 96-well plates at 4,000 cells per well. The following day, 100 μL of medium containing varying concentrations of W-1-32 (0, 3.125, 6.25, 12.5, 25, 50, 100, and 200 μM) was added. Cells were treated for 24, 48, and 72 h. At each time point, 10 μL of CCK-8 reagent was added to each well, followed by incubation at 37 °C. Absorbance was measured at 450 nm using a microplate reader (SpectraMax, M5).

### 2.8 Wound-healing assays and transwell assays

Eca109 and KYSE150 cells were seeded in 6-well plates at 1 × 10^6^ cells per well and incubated for 24 h. Once cell density reached 90%, the monolayer was scratched using a sterile 20 μL micropipette tip, followed by three gentle washes with PBS. Serum-free medium (2 mL) containing varying concentrations of W-1-32 (0, 7.5, 15, and 30 μM) was then added. Images of the same field were captured at 0, 24, and 48 h using a microscope, and the wound area was analyzed with ImageJ. The wound healing rate was calculated as follows:

$$\text{Woundhealingrate}=\left(\frac{\text{woundwidthat}\;0\;h-\text{wound}\;\text{width}\;\text{at}\;48\;h}{\text{wound}\;\text{width}\;\text{at}\;0\;h}\right)\times 100\%$$

Tumor cell migration assays were conducted in 24-well plates using transwell chambers with 8 μm pores. Eca109 and KYSE150 cells were suspended in serum-free RPMI-1640 medium at 2.5 × 10^6^ cells/mL. A 200 μL cell suspension was added to the upper chamber, while 500 μL of medium containing varying concentrations of W-1-32 was added to the lower chamber. The plate was incubated at 37 °C for 24 h, after which the chamber was washed with PBS, and non-migrated cells in the upper chamber were removed. The migrated cells were fixed with 4% formaldehyde for 30 min, stained with 0.1% crystal violet, and imaged under a microscope. ImageJ was used for quantification.

### 2.9 Quantitative real-time polymerase chain reaction (qRT-PCR)

The PrimeScript™ RT Reagent Kit (Feijie, China) was used for reverse transcription of total RNA. Genomic DNA was removed at 42 °C for 2 min using gDNA Eraser. Total RNA from ESCC cells was then reverse transcribed into cDNA with 5× HiScript III qRT Super Mix under the following conditions: 37 °C for 15 min, followed by 85 °C for 5 s. The product was immediately stored at −20 °C until use. qRT-PCR was performed on a QuantStudio 5 Real-Time PCR System (Applied Biosystems, Foster City, USA) under the following conditions: 95 °C for 30 s, followed by 40 cycles of 95 °C for 5 s and 60 °C for 1 min. Relative quantification was determined using the 2^−ΔΔCT^ method, normalized to GAPDH.

Primer sequence for XIAP:

Forward primer sequence (5′–3′) AAT​AGT​GCC​ACG​CAG​TCT​ACA.

Reverse primer sequence (5′–3′) CAG​ATG​GCC​TGT​CTA​AGG​CAA.

### 2.10 Western blot analysis

All cells were lysed using RIPA buffer with a protease inhibitor. Protein concentrations were normalized using the bicinchoninic acid assay kit. Lysates were separated by Sodium dodecyl-sulfate polyacrylamide gel electrophoresis and transferred to Nitrocellulose membranes (GE, USA). The membranes were blocked with 5% skim milk for 2 h at room temperature, then incubated overnight at 4 °C with primary antibodies against XIAP (1:1,000) and GAPDH (1:5,000). Bands were visualized using enhanced chemiluminescence reagent (UE, China).

#### 2.10.1 Molecular docking

Molecular docking simulations were performed using AutoDock 4.2 (https://autodock.scripps.edu/download-autodock4/) with AutoDockTools1.5.6 (https://ccsb.scripps.edu/mgltools/downloads/) to investigate the binding mode of the ligand within the receptor binding pocket. The receptor and ligand structures were prepared in PDBQT format, with polar hydrogens added and Gasteiger charges assigned prior to docking. A three-dimensional grid box was generated to encompass the target binding site, with grid dimensions of 94 × 62 × 74 points along the x, y, and z-axes and a grid spacing of 0.375 Å. The grid center was set at (−17.306, −19.536, −4.319) Å. Docking poses were ranked based on predicted binding free energy, and the lowest-energy conformations were selected for further analysis. Protein–ligand interaction patterns, including hydrogen bonds and hydrophobic contacts, were visualized and analyzed using LigPlot+ (v2.3.0, https://www.ebi.ac.uk/thornton-srv/software/LigPlus/) and UCSF Chimera (version 1.18, https://www.cgl.ucsf.edu/chimera/).

#### 2.10.2 Molecular dynamics simulations and trajectory analysis

All-atom molecular dynamics (MD) simulations were performed using OpenMM (v8.0, https://github.com/openmm/openmm) with GPU acceleration (NVIDIA TESLA v100 16GB). The protein–ligand complex was constructed from a pre-equilibrated PDB structure, with the ligand parameterized using a custom force field file. The AMBER99SB force field was applied to the protein, TIP3P was used for explicit solvation, and the system was solvated in a periodic water box with a padding distance of 1.0 nm. Long-range electrostatic interactions were treated using the particle mesh Ewald (PME) method with a real-space cutoff of 1.0 nm, and all covalent bonds involving hydrogen atoms were constrained. Simulations were carried out on a CUDA-enabled GPU (mixed precision), with PME calculations performed on the GPU.

MD simulations were performed under the NVT ensemble at 300 K using a Langevin integrator with a friction coefficient of 1 ps^-1^ and a time step of 2 fs. The production simulation was run for 50 million steps. Trajectories were recorded every 100,000 steps in DCD format, and thermodynamic properties were monitored every 10,000 steps. Trajectory analyses were carried out using MDTraj. Root-mean-square deviation (RMSD) and root-mean-square fluctuation (RMSF) were calculated for the protein, ligand, and complex. Hydrogen bonds between the protein and ligand were identified using the Baker–Hubbard algorithm. The radius of gyration (Rg) was computed to assess global structural compactness. Two- and three-dimensional free energy landscapes were constructed from RMSD-based collective variables using Boltzmann inversion of probability distributions at 300 K. All analysis data were exported in CSV format for further processing and visualization.

#### 2.10.3 Well-tempered metadynamics simulations

WT-MetaD simulations were carried out under the NVT ensemble at 300 K using a Langevin integrator with a friction coefficient of 1 ps^-1^ and a time step of 2 fs. A total of five independent WT-MetaD runs were performed with distinct random seeds applied to the integrator to improve statistical sampling. The collective variable (CV) was defined as the distance between the mass-weighted centers of mass of the protein and ligand. Gaussian bias potentials were deposited every 5,000 steps with an initial height of 1.2 kJ mol^-1^ and a width of 0.2 nm. A bias factor (γ) of 15 was used, corresponding to a bias temperature of ΔT = (γ − 1)T. The time-dependent bias potential and CV values were recorded throughout the simulations and used for subsequent free energy reconstruction and convergence analysis.

### 2.11 Statistical analysis

All data are presented as means ± standard deviation (SD) from at least three independent experiments. Statistical analyses and image generation were performed using GraphPad Prism (version 8.0). The students’ unpaired t-test was used for pairwise comparisons, while a one-way analysis of variance (ANOVA) with a *post hoc* test was applied for multiple-group comparisons. All bioinformatics analyses and visualizations were performed using R software (version4.2.0) and OmicsTools analysis software (https://github.com/zihaoxingstudy1/). P < 0.05 was considered statistically significant.

## 3 Results

### 3.1 Pan-cancer analysis of XIAP

**FIGURE 1:**
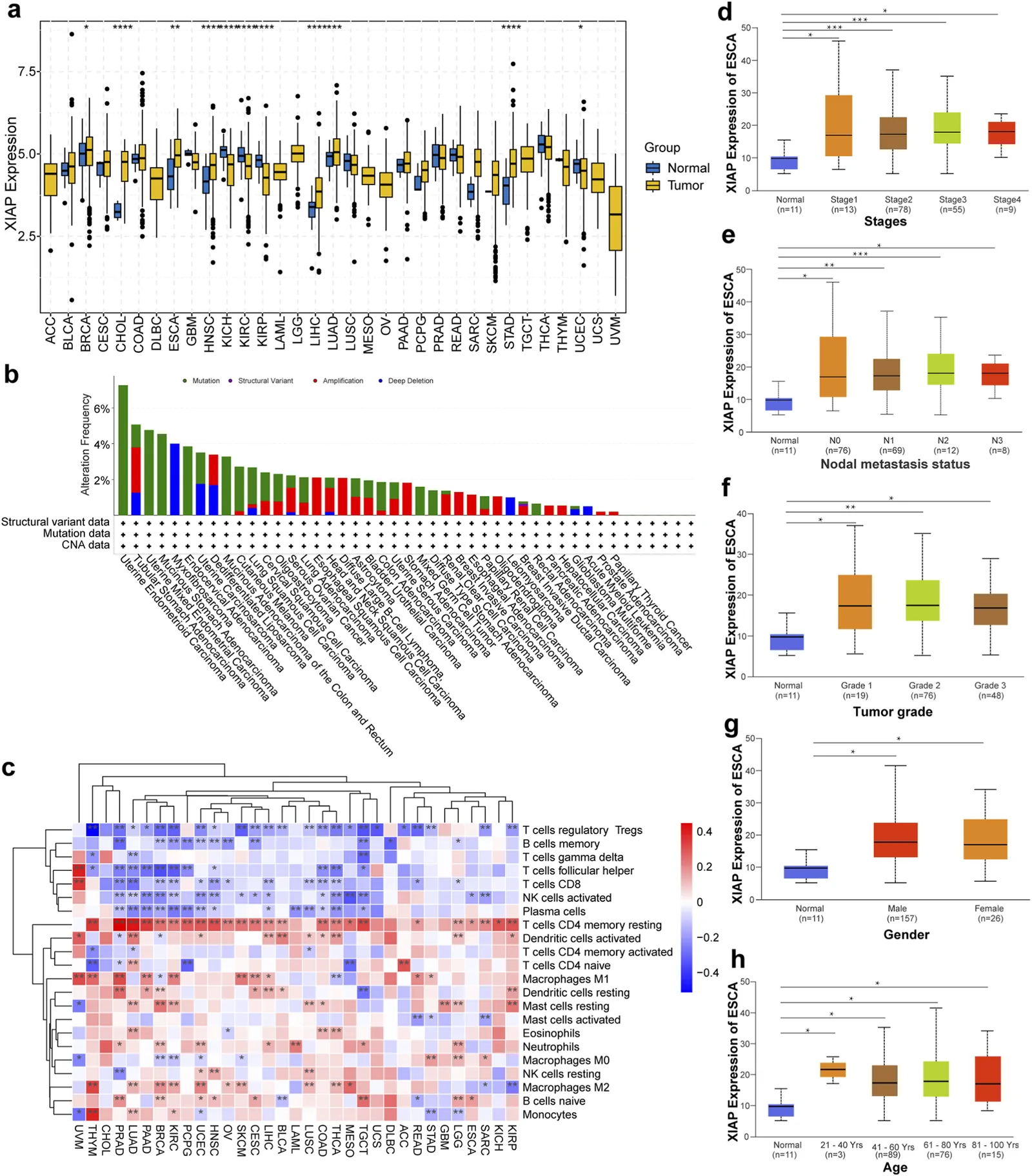
Pan-cancer analysis of XIAP. **(a)** Differential expression of XIAP between tumor and normal tissues across various cancer types. **(b)** Genetic alteration analysis of XIAP across different cancers. **(c)** Correlation analysis between XIAP expression and immune cell infiltration. **(d–h)** Expression trends of XIAP in esophageal carcinoma stratified by stage **(d)**, lymph node metastasis status **(e)**, grade **(f)**, gender **(g)**, and age **(h)**.

To evaluate the clinical relevance of XIAP in cancer, we first performed a pan-cancer analysis using TCGA datasets. XIAP expression was significantly upregulated in 11 cancer types, including bladder urothelial carcinoma (BLCA), cholangiocarcinoma (CHOL), esophageal carcinoma (ESCA), and head and neck squamous cell carcinoma (HNSC), compared with adjacent normal tissues (Figure 1a). Among these, ESCA exhibited pronounced XIAP overexpression, suggesting a potential oncogenic role in esophageal tumorigenesis. Analysis of genetic alterations via the cBioPortal platform revealed that XIAP mutation was the predominant alteration type. In ESCA, amplification was the primary alteration, occurring at a frequency of 2% (Figure 1b). Given the established role of the tumor microenvironment in ESCC progression, we next examined the correlation between XIAP expression and immune cell infiltration. XIAP expression was positively correlated with mast cells and macrophages, while negatively correlated with cytotoxic cells, CD8^+^ T cells, and natural killer (NK) cells (Figure 1c). Notably, in ESCA, XIAP expression showed a significant negative correlation with CD8^+^ T cells and activated NK cells, indicating that XIAP may function as an immunosuppressive molecule in esophageal carcinoma. Stratified analysis of ESCA samples based on clinicopathological characteristics further demonstrated that XIAP expression was significantly elevated across all subgroups, including different ages, genders, tumor stages, grades, and lymph node metastasis statuses,(Figures 1d–h). XIAP expression was positively associated with clinical stage, and gradually elevated along with tumor progression. Collectively, these findings highlight XIAP as a potential biomarkers in various tumors, supporting its candidacy as a therapeutic target.

### 3.2 Expression, prognostic value and cellular distribution of XIAP in ESCC

**FIGURE 2:**
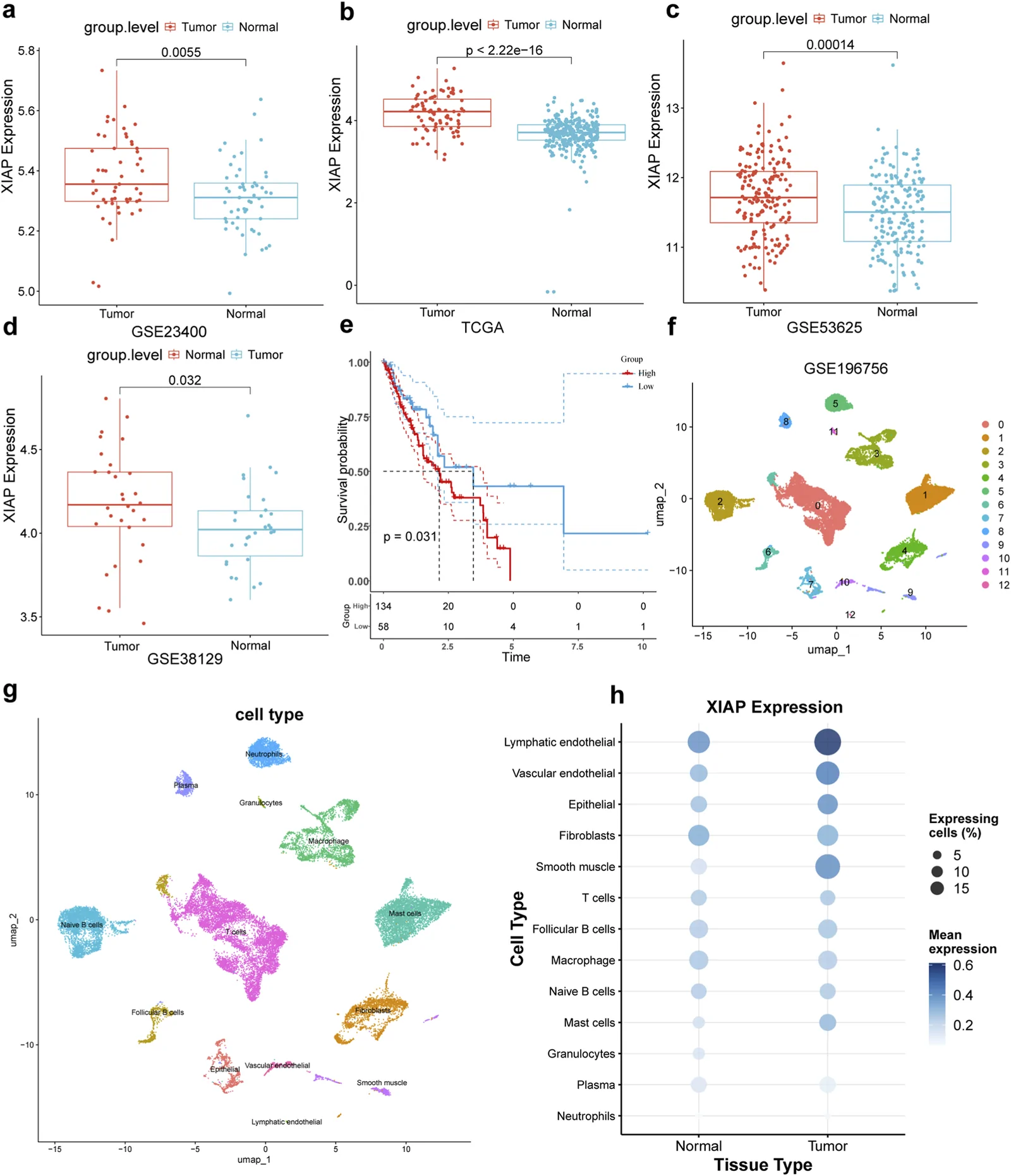
Expression, prognostic value and cellular distribution of XIAP in ESCC. **(a–d)** XIAP expression levels in tumor versus adjacent normal tissues from four independent GEO datasets: GSE23400, TCGA, GSE53625, GSE38129. **(e)** Kaplan–Meier survival analysis of XIAP expression in TCGA esophageal cancer cohort. **(f)** A umap plot showing 13 distinct cell clusters identified from the GSE196756 single-cell RNA sequencing dataset. **(g)** A umap plot displaying the classification of cell types. **(h)** Bubble plot showing the expression distribution of XIAP in 13 cell subpopulations.

To further validate the expression pattern of XIAP in esophageal cancer, we analyzed four independent GEO datasets. Consistently, XIAP expression was significantly upregulated in tumor tissues compared with adjacent normal tissues across all datasets (Figures 2a–d). Survival analysis using the TCGA esophageal cancer cohort revealed that patients with high XIAP expression exhibited significantly worse overall survival than those with low expression, highlighting its prognostic significance (Figure 2e).

To dissect the cellular distribution of XIAP within the tumor microenvironment, we performed single-cell RNA sequencing analysis using the GSE196756 dataset. Unsupervised clustering identified 13 distinct cell clusters (Figure 2f). A total of 13 cell subpopulations were identified (Figure 2g). XIAP expression was predominantly enriched in epithelial and endothelial cell clusters (Figure 2h). The specific enrichment of XIAP in epithelial cells aligns with our previous findings that XIAP promotes esophageal cancer cell migration and invasion by inducing epithelial-mesenchymal transition (EMT), further supporting its functional role in ESCC progression.

### 3.3 Functional enrichment analysis of XIAP high-expression signature in ESCC

**FIGURE 3:**
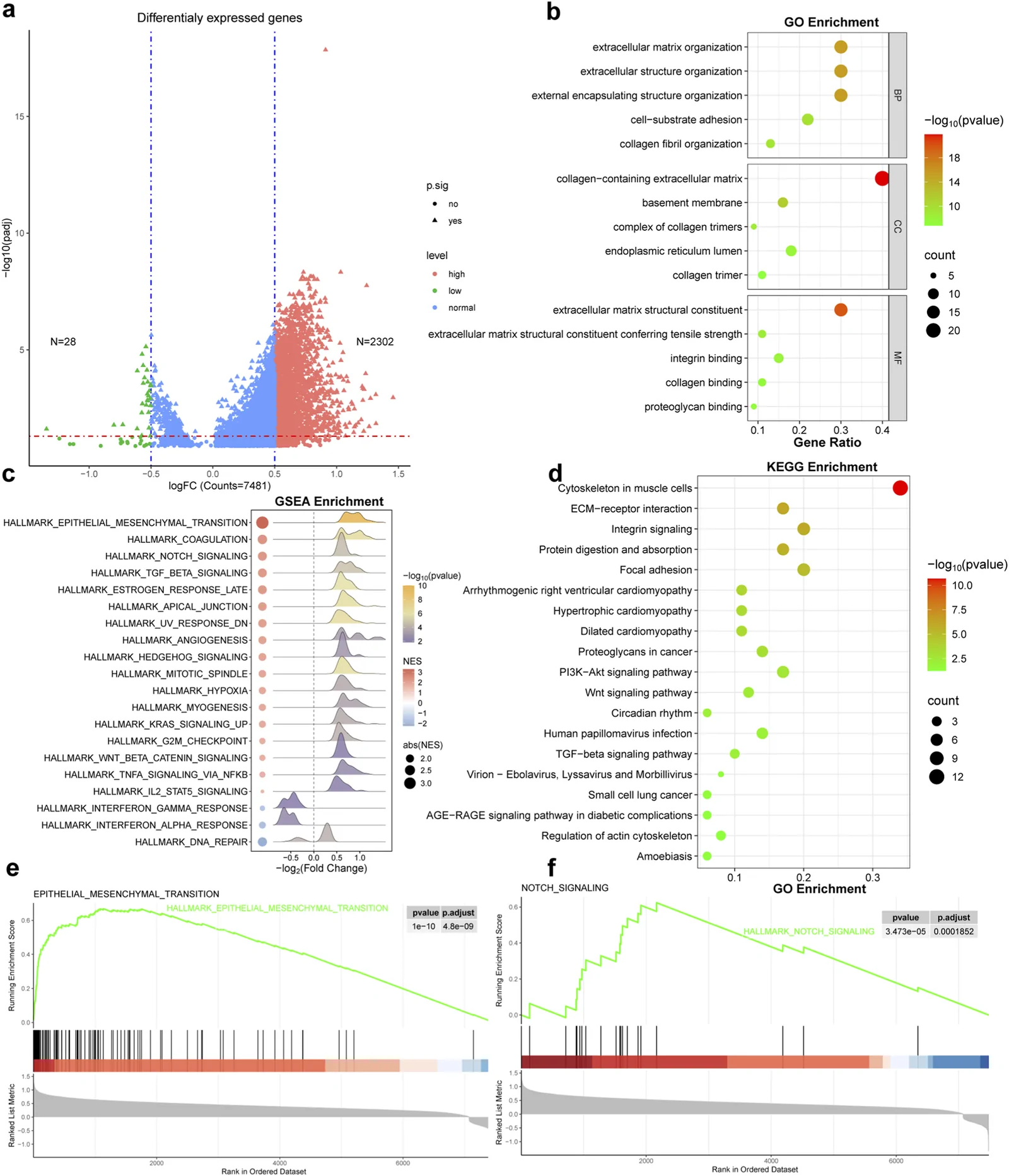
Functional enrichment analysis of XIAP high-expression signature in ESCC. **(a)** Volcano plot showing differentially expressed genes between XIAP-high and XIAP-low groups in the TCGA ESCC cohort. **(b)** Gene Ontology (GO) enrichment analysis of upregulated genes in XIAP-high ESCC samples, including biological processes (BP), cellular components (CC), and molecular functions (MF). **(c)** Gene Set Enrichment Analysis (GSEA) of Hallmark gene sets comparing XIAP-high versus XIAP-low groups. The normalized enrichment score (NES) and adjusted p-value are shown for selected pathways. **(d)** KEGG pathway enrichment analysis of upregulated genes in XIAP-high ESCC samples. **(e)** Enrichment plot of the Hallmark Epithelial-Mesenchymal Transition (EMT) pathway from GSEA. **(f)** Enrichment plot of the Hallmark Notch signaling pathway from GSEA.

To investigate the biological processes associated with XIAP overexpression in esophageal squamous cell carcinoma, we performed differential expression analysis between XIAP-high and XIAP-low groups in the TCGA ESCC cohort. A total of 2,302 upregulated genes and 28 downregulated genes were identified (Figure 3a).

Gene Ontology (GO) enrichment analysis revealed that the upregulated genes were predominantly enriched in biological processes related to extracellular matrix organization, extracellular structure organization, and cell-substrate adhesion (Figure 3b). In terms of cellular components, these genes were significantly associated with the collagen-containing extracellular matrix, basement membrane, and collagen trimer complexes. Molecular function analysis further indicated enrichment in extracellular matrix structural constituent, integrin binding, and collagen binding (Figure 3b). Consistent with these findings, KEGG pathway analysis showed significant enrichment in pathways such as cytoskeleton in muscle cells, ECM-receptor interaction, integrin signaling, focal adhesion, and protein digestion and absorption (Figure 3d). Additionally, pathways including PI3K-Akt, Wnt, and TGF-beta signaling were also enriched, suggesting multifaceted roles of XIAP in tumor progression.

To further delineate the signaling pathways associated with XIAP high expression, we performed Gene Set Enrichment Analysis (GSEA) using the Hallmark gene sets. Notably, the Epithelial–Mesenchymal Transition (EMT) pathway was the most significantly enriched, with a normalized enrichment score (NES) of 3.29 (Figure 3c). Other positively enriched pathways included Coagulation, Notch signaling, TGF-beta signaling, Apical junction, and Angiogenesis, while DNA repair and interferon responses were negatively enriched (Figure 3c). Visualization of the leading edge enrichment confirmed the strong association between XIAP high expression and the EMT (Figure 3e) and Notch signaling (Figure 3f) pathways. These results demonstrate that XIAP high expression in ESCC is closely associated with extracellular matrix remodeling, cell adhesion, and key oncogenic signaling pathways, particularly EMT and Notch signaling, which are known to promote tumor invasion and metastasis.

### 3.4 W-1-32 is identified as a novel XIAP inhibitor

**FIGURE 4:**
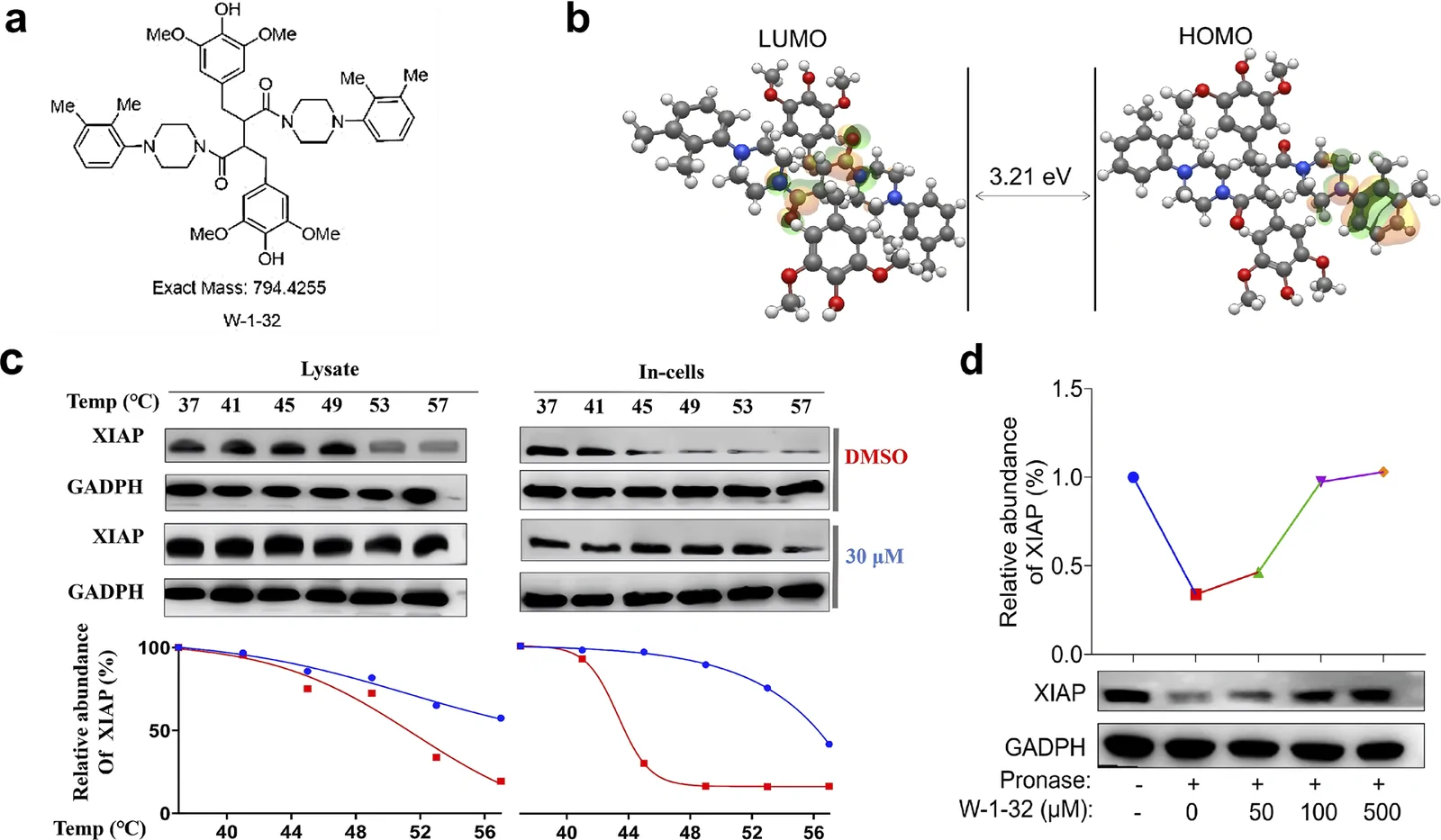
W-1-32 directly binds to XIAP in KYSE150 cells. **(a)** Chemical structure of W-1-32. **(b)** LUMO and HOMO of W-1-32 calculated by density functional theory (DFT) calculations. **(c)** Cellular thermal shift assay (CETSA) showing the interaction between W-1-32 and XIAP in intact KYSE150 cells and cell lysates. **(d)** Drug affinity responsive target stability (DARTS) assay performed in KYSE150 cell lysates to validate direct binding of W-1-32 to XIAP.

Our previous studies demonstrated a significant correlation between high XIAP expression and lymph node metastasis in ESCC (Jin et al., 2019). Additionally, high XIAP expression was identified as an independent risk factor for poor prognosis, with significantly lower overall survival in ESCC patients compared to those with low expression. Given the clinical significance of targeting XIAP, we aimed to identify novel XIAP inhibitors with high efficacy against ESCC cells. To achieve this, we screened a series of natural products and their structural derivatives, and ultimately identified a novel small-molecule compound, W-1-32 as the potential candidate (chemical structure depicted in Figure 4a). W-1-32 is a symmetric, multifunctional small molecule composed of phenolic aromatic rings, amide linkages, and piperazine units, combining rigid aromatic frameworks with flexible heterocyclic segments. Redox-active phenolic hydroxyls together with multiple hydrogen-bonding sites and protonatable piperazine nitrogens confer amphiphilicity and enable versatile molecular interactions.

### 3.5 Electronic structure and reactivity analysis of W-1-32

DFT calculations were performed to elucidate the electronic properties and reactivity profile of W-1-32. As summarized in Supplementary Table S1, the molecule exhibits a HOMO energy of −5.33 eV and a LUMO energy of −2.12 eV, resulting in a HOMO–LUMO gap of 3.21 eV. This moderate energy gap indicates a balance between electronic stability and chemical reactivity, consistent with a molecule capable of engaging in controlled electronic interactions under biological conditions.

Visualization of the frontier molecular orbitals (Figure 4b) reveals that the HOMO is predominantly delocalized over the phenolic aromatic moieties, with significant contribution from the hydroxyl-substituted rings. This spatial distribution suggests that the phenolic groups act as primary electron-donating centers, supporting the ability of W-1-32 to participate in hydrogen atom transfer or single-electron transfer processes. In contrast, the LUMO is mainly localized on the central scaffold and carbonyl-containing regions, indicating that these moieties are preferentially involved in electron acceptance and polarization interactions during molecular recognition.

Consistent with these orbital features, conceptual DFT descriptors derived from frontier orbital energies further characterize the reactivity of W-1-32. As given in Supplementary Table S1, the calculated chemical hardness (η = 1.61 eV) and softness (S = 0.31 eV^-1^) reflect moderate resistance to charge redistribution, while maintaining sufficient flexibility for electronic adaptation upon binding. The relatively high electronegativity (χ = 3.72 eV) and chemical potential (μ = −3.72 eV) indicate a strong tendency to attract electron density, whereas the electrophilicity index (ω = 4.31 eV) suggests appreciable but controlled electrophilic character.

Collectively, the HOMO/LUMO distributions and global reactivity descriptors indicate that W-1-32 possesses a well-balanced electronic architecture, combining stable aromatic electron-donating domains with polar acceptor regions.

### 3.6 Direct target engagement of W-1-32 with XIAP

Based on the principle that ligand binding increases protein thermal stability, cellular thermal shift assays (CETSA) were performed to examine the interaction between W-1-32 and XIAP. As shown in Figure 4c, W-1-32 markedly enhanced the thermal stability of XIAP in both KYSE150 cell lysates and intact cells. In cell lysates, XIAP underwent pronounced thermal denaturation at temperatures ≥53 °C in the DMSO control, whereas W-1-32 treatment significantly attenuated XIAP degradation, preserving approximately 60% of the protein. To further validate direct target engagement, a drug affinity responsive target stability (DARTS) assay was conducted. As shown in Figure 4d, W-1-32 dose-dependently protected XIAP from pronase-mediated proteolysis in KYSE150 cell lysates. Collectively, these results demonstrate that W-1-32 directly binds to XIAP and enhances its structural stability in both cellular and cell-free systems.

### 3.7 W-1-32 suppresses the proliferation and migration of ESCC cells

**FIGURE 5:**
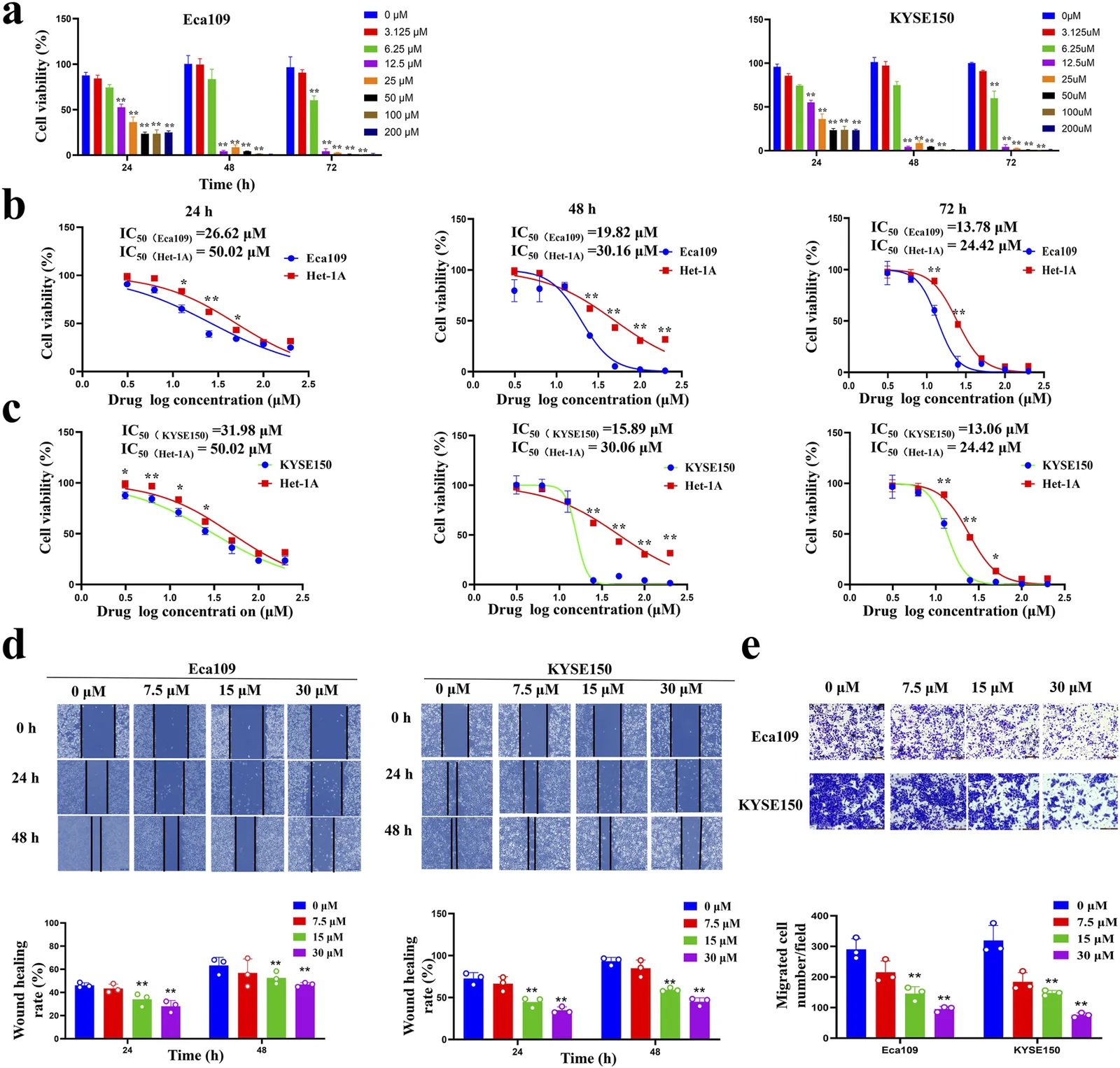
W-1-32 suppresses proliferation and migration of ESCC cells. **(a)** Inhibition of Eca109 and KYSE150 cell proliferation by W-1-32. **(b,c)** Proliferation of Eca109 and KYSE150 cells following treatment with W-1-32 for 24, 48 h and 72 h as assessed by cell viability assays. **(d)** Cell migration of Eca109 and KYSE150 cells after W-1-32 treatment for 24 and 48 h, evaluated using a wound-healing assay. The black line indicates the wound boundary. **(e)** Migration and invasion of Eca109 and KYSE150 cells assessed using Transwell assays following W-1-32 treatment. Data are presented as mean ± SD. *P < 0.05, **P < 0.01.

**FIGURE 6:**
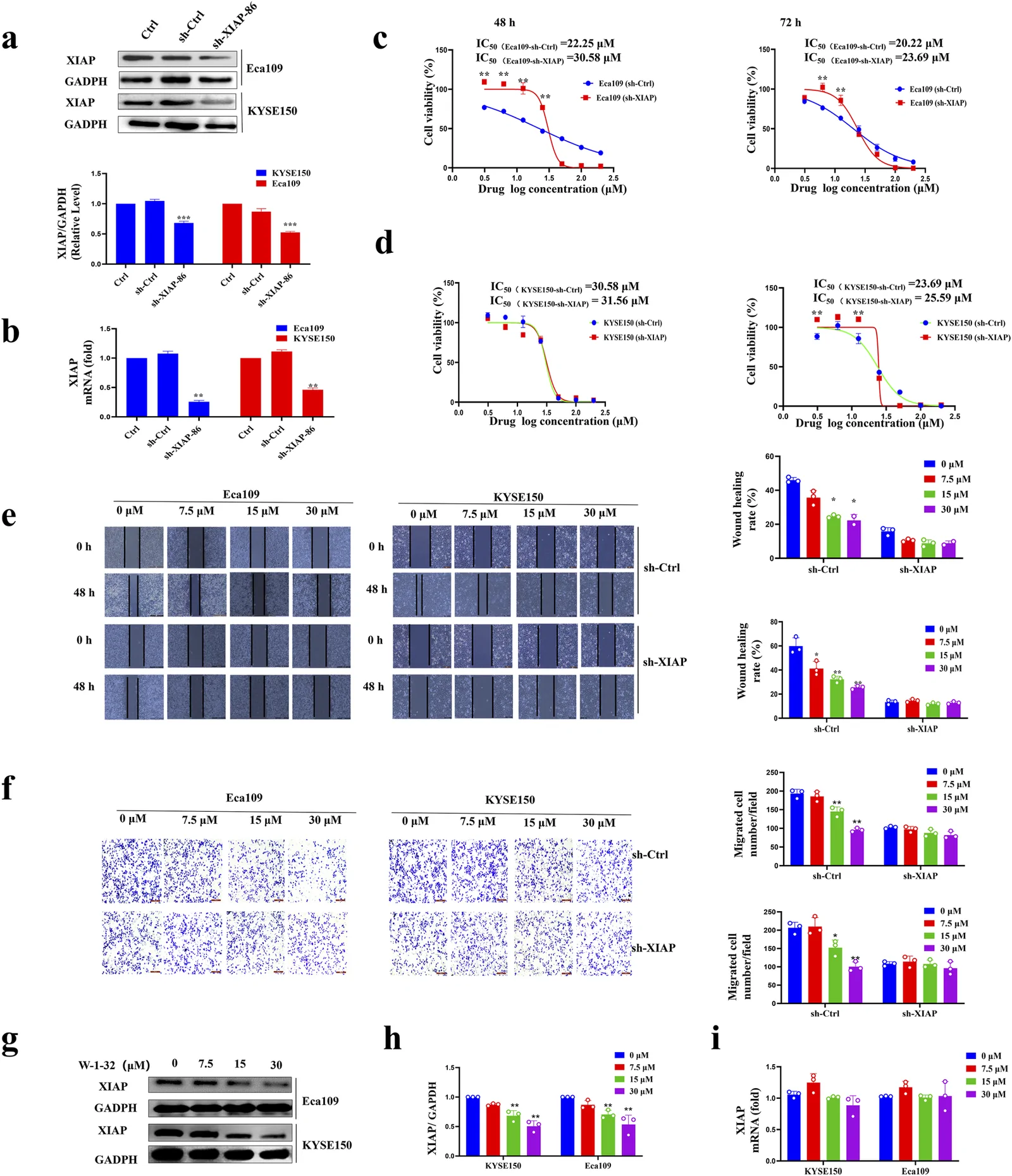
W-1-32 inhibits ESCC cell migration and invasion in an XIAP-dependent manner. **(a,b)** Generation of stable XIAP-knockdown ESCC cell lines. XIAP expression in sh-Ctrl and sh-XIAP cells was validated by immunoblotting and qRT–PCR. **(c,d)** Dose–response curves and half-maximal inhibitory concentration (IC_50_) values of W-1-32 in Eca109 and KYSE150 cells with or without XIAP knockdown, as determined by CCK-8 assays following treatment for 48 and 72 h. **(e)** Wound healing assays evaluating the migration abilities of Eca109 and KYSE150 cells in sh-Ctrl and sh-XIAP groups after treatment with W-1-32 (0, 7.5, 15, 30 μM). Representative images of the same scratch area were captured at 0 h and 48 h post-scratching. **(f)** Transwell assays assessing the migration abilities of Eca109 and KYSE150 cells in sh-Ctrl and sh-XIAP groups following treatment with different concentrations of W-1-32 (0, 7.5, 15, 30 μM). Representative images and quantitative analysis of migrated cell counts are shown. **(g,h)** The expression of XIAP protein levels in Eca109 and KYSE150 cells treated with W-1-32 were detected by Western blot. **(i)** The expression of mRNA in Eca109 and KYSE150 cells treated with W-1-32 were detected by qRT-PCR. Data are presented as mean ± SD. *P < 0.05, **P < 0.01.

**FIGURE 7:**
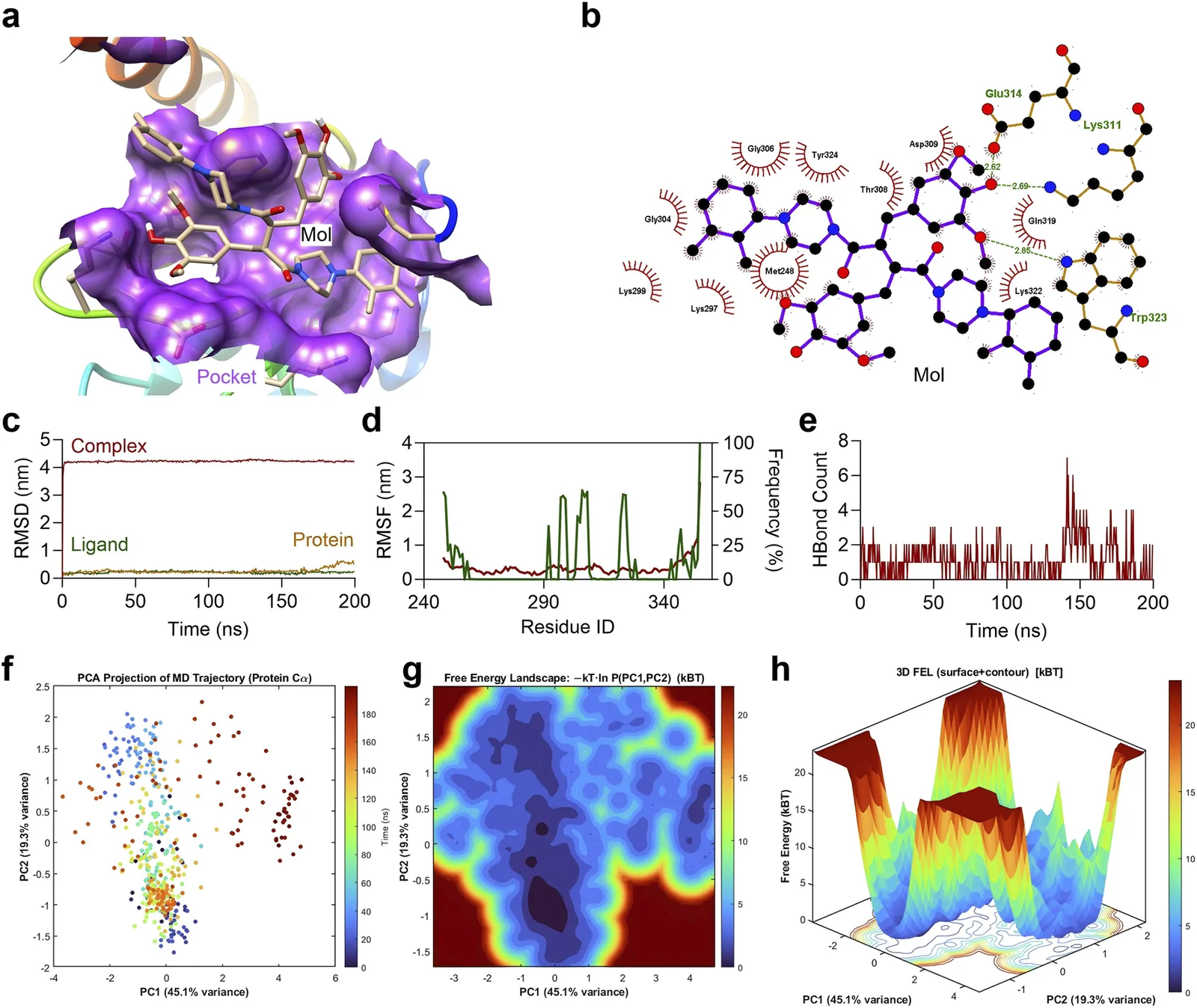
Molecular docking and molecular dynamics simulations reveal the binding mode of W-1-32 to XIAP. **(a)** Predicted binding pose of W-1-32 within the active pocket of XIAP. The protein surface highlights the binding cavity by purple color. **(b)** Two-dimensional interaction diagram of the XIAP–W-1-32 complex generated using LigPlot+, showing hydrogen bonds (green dashed lines) and nonbonded contacts (red arcs). **(c)** RMSD profiles of the XIAP–W-1-32 complex, protein backbone, and ligand over the 200-ns molecular dynamics simulation. **(d)** RMSF of XIAP residues and corresponding interaction frequencies with W-1-32 during the 200-ns simulation. **(e)** Time evolution of hydrogen bond formation between XIAP and W-1-32 throughout the simulation. **(f)** Principal component analysis (PCA) of the XIAP–W-1-32 complex based on the 200-ns trajectory. **(g,h)** Two-dimensional and three-dimensional free energy landscapes (FELs) of the XIAP–W-1-32 complex derived from the molecular dynamics simulation.

**FIGURE 8:**
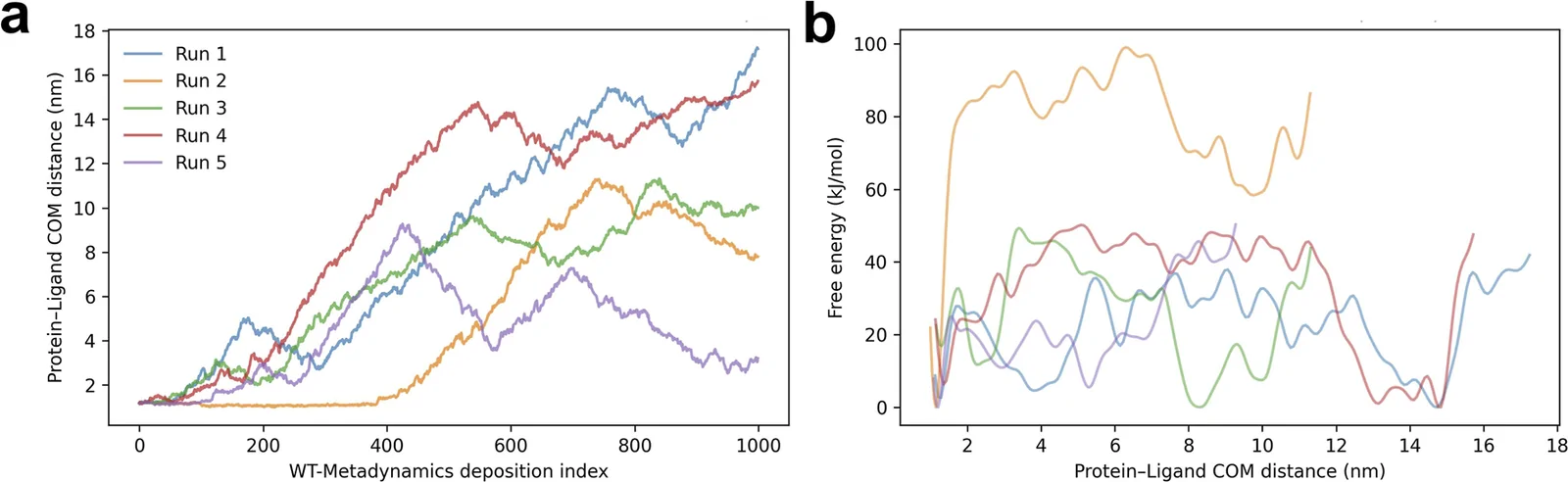
Ligand egress behavior and free-energy profiles revealed by well-tempered metadynamics. **(a)** Time evolution of the protein–ligand center-of-mass (COM) distance from five independent WT-metadynamics simulations performed with different random seeds. **(b)** Corresponding free-energy profiles reconstructed along the COM distance.

Following confirmation of direct binding between W-1-32 and XIAP, we next evaluated the antitumor activity of W-1-32 in esophageal squamous cell carcinoma (ESCC) cells. Cell viability assays were performed in two ESCC cell lines with high XIAP expression (Eca109 and KYSE150), as well as in normal human esophageal epithelial cells (Het-1A). As shown in Figures 5a,b W-1-32 inhibited the proliferation of Eca109 and KYSE150 cells in a concentration- and time-dependent manner at both 24 and 48 h, whereas Het-1A cells exhibited markedly lower sensitivity. At 24 h, the IC_50_ values of W-1-32 were 26.62 μM for Eca109 and 19.82 μM for KYSE150 cells, while the IC_50_ for Het-1A cells exceeded 50 μM. A similar selective inhibitory profile was observed at 48 h, with IC_50_ values of 31.98 μM (KYSE150) and 15.89 μM (Eca109), compared with substantially higher IC_50_ values in Het-1A cells (Figures 5b,c). These results indicate that W-1-32 preferentially suppresses ESCC cell proliferation while sparing normal esophageal epithelial cells. Based on these findings, concentrations of 7.5, 15, and 30 μM were selected for subsequent functional assays. Wound-healing assays revealed that W-1-32 significantly impaired the migratory capacity of both Eca109 and KYSE150 cells in a dose-dependent manner at 24 and 48 h (Figure 5d). Consistently, Transwell migration assays demonstrated a marked reduction in the number of migrating ESCC cells following W-1-32 treatment (Figure 5e). Collectively, these results demonstrate that W-1-32 effectively suppresses ESCC cell proliferation and migration *in vitro*.

### 3.8 XIAP contributes to the cellular response to W-1-32 in ESCC cells

To examine whether the antiproliferative effects of W-1-32 are dependent on XIAP, stable XIAP-knockdown ESCC cell lines were generated. Efficient depletion of XIAP in both Eca109 and KYSE150 cells was confirmed at the protein and mRNA levels by immunoblotting and qRT–PCR analyses (Figures 6a,b). The sensitivity of ESCC cells to W-1-32 was then evaluated following XIAP knockdown. As shown in Figures 6c,d, dose–response analyses revealed that silencing XIAP altered the growth-inhibitory response to W-1-32 in both cell lines. In Eca109 cells, XIAP knockdown resulted in increased IC_50_ values at 48 h and 72 h compared with sh-Ctrl cells, indicating reduced sensitivity to W-1-32. A similar trend was observed in KYSE150 cells, where XIAP depletion led to a modest attenuation of W-1-32–mediated growth inhibition across multiple time points. Collectively, these data indicate that the antiproliferative activity of W-1-32 in ESCC cells is, at least in part, dependent on XIAP expression, supporting XIAP as a functional target contributing to the cellular response to W-1-32.

### 3.9 Inhibitory effects of W-1-32 on ESCC cell migration and invasion

Our previous studies demonstrated that XIAP knockdown plays a crucial role in ESCC cell proliferation and migration (Jin et al., 2019). The effects of W-1-32 on ESCC cell migration and invasion were further evaluated in Eca109 and KYSE150 cells with or without XIAP knockdown. As shown in Figure 6e, wound-healing assays demonstrated that W-1-32 reduced the migratory capacity of both ESCC cell lines in a concentration-dependent manner at 48 h. This inhibitory effect was evident in sh-Ctrl cells and was attenuated in sh-XIAP cells, indicating reduced sensitivity to W-1-32–mediated migration suppression following XIAP depletion. Consistent with these findings, Transwell assays revealed a significant decrease in the number of migrated and invaded cells after W-1-32 treatment (Figure 6f). The extent of migration and invasion inhibition was more pronounced in sh-Ctrl cells than in sh-XIAP cells in both Eca109 and KYSE150 lines. Together, these results indicate that W-1-32 suppresses ESCC cell migratory and invasive behavior *in vitro*, with the magnitude of inhibition partially influenced by XIAP expression.

Finally, the effects of W-1-32 on XIAP expression were examined at both the protein and mRNA levels in ESCC cells. As shown in Figures 6g,h, treatment with W-1-32 for 24 h resulted in a concentration-dependent reduction of XIAP protein levels in both Eca109 and KYSE150 cells. In contrast, quantitative real-time PCR analysis revealed no significant changes in XIAP mRNA expression following W-1-32 treatment (Figure 6i). These findings indicate that W-1-32 reduces XIAP protein abundance without affecting its transcription, suggesting a post-translational modification mode of regulation associated with the cellular response to W-1-32.

### 3.10 Docking and molecular dynamics analysis of W-1-32 binding to XIAP

To elucidate the molecular basis of the interaction between W-1-32 and XIAP, molecular docking followed by MD simulations was performed. As shown in Figure 7a W-1-32 was predicted to occupy a well-defined binding pocket of XIAP, adopting a stable pose that is accommodated by the surrounding cavity. Two-dimensional interaction analysis (Figure 7b) indicated that W-1-32 engages in multiple hydrogen-bonding and nonbonded interactions with residues located within the binding region, including residues Met248, Lys297, Lys 299, Gly304, Thr308, Asp309, Gln319, Lys322, and Tyr324. Additionally, hydrogen bond between XIAP and W-1-32 were formed, including Lys311, Glu314, Trp323, suggesting a network of polar and hydrophobic contacts contributing to ligand recognition.

Molecular electrostatic potential (MEP) analysis revealed a pronounced global minimum (Vmin = −0.097 a.u.) localized near the phenolic oxygen atom O26 (Supplementary Figure S1). The minimum potential was located at (−1.182, 1.359, −1.838 Å), within 1.26 Å of O26, identifying this site as the dominant electron-rich region of W-1-32. This region coincides with the hydrogen-bond-accepting environment of the XIAP binding pocket and is spatially proximal to pocket residues 248, 297, 299, 304, 308, and 309 identified in docking and molecular dynamics analyses (Figure 7b). Conversely, the global maximum electrostatic potential (Vmax = 183.76 a.u.) was detected adjacent to atom C58 (0.02 Å), a carbon atom associated with the protonatable piperazine moiety, defining the principal electropositive surface of the molecule. This electropositive region is oriented toward acidic and polar residues within the XIAP pocket, including residues 311, 314, 319, 322, 323, and 324. Collectively, the spatial separation of MEP extrema delineates a polarized electrostatic surface that is consistent with the stable XIAP–W-1-32 binding mode observed in molecular dynamics simulations and supports ligand engagement through coordinated electrostatic and hydrogen-bond interactions.

The stability of the XIAP–W-1-32 complex was further evaluated by a 200-ns MD simulation. RMSD analysis demonstrated that the protein backbone, ligand, and complex rapidly reached equilibrium and remained stable throughout the simulation (Figure 7c). Consistently, RMSF profiling revealed limited fluctuations of most residues, with interaction-associated residues exhibiting relatively low mobility (Figure 7d), while frequency of non-covalent contacts was consistent with RMSF results. Hydrogen bond analysis showed persistent intermolecular hydrogen bonding between XIAP and W-1-32 over the simulation period (Figure 7e), supporting sustained ligand engagement.

Principal component analysis (PCA) of the MD trajectory indicated a confined conformational space for the XIAP–W-1-32 complex (Figure 7f). Correspondingly, free energy landscape (FEL) analyses revealed the presence of well-defined low-energy basins, consistent with a stable binding mode during the simulation (Figures 7g,h). Collectively, these computational results are consistent with a stable binding mode between W-1-32 and XIAP.

### 3.11 Ligand egress behavior and free-energy landscape revealed by WT-metadynamics

To investigate the reproducibility and energetic features of ligand dissociation, we performed five independent well-tempered metadynamics (WT-MetaD) simulations using the protein–ligand center-of-mass (COM) distance as the collective variable. As shown in Figure 8a, all simulations exhibited a qualitatively similar time evolution of the COM distance, demonstrating reproducible ligand egress behavior despite different initial velocity seeds. In each run, the ligand initially remained in close proximity to the binding pocket, corresponding to a stable bound state, before undergoing a pronounced increase in the COM distance that marked the onset of ligand unbinding.

Although the exact timing of the egress event varied among independent trajectories, the overall dissociation pattern was conserved across all runs. Following egress, the ligand sampled a broad range of COM distances, consistent with diffusion in the solvent-exposed region. The absence of sustained rebinding to the initial bound state after dissociation indicates that the applied bias efficiently drove the system across the dominant free-energy barrier associated with ligand unbinding.

Reconstruction of the free-energy profiles along the egress coordinate further corroborated these observations (Figure 8b). Independent WT-MetaD runs yielded comparable free-energy landscapes, characterized by a distinct minimum corresponding to the bound state and a finite free-energy barrier separating the bound and dissociated regions. In addition, shallow intermediate features were observed along the dissociation pathway, suggesting the presence of transient metastable states during ligand exit. The consistency of the free-energy profiles across multiple simulations supports the robustness of the identified ligand egress mechanism and indicates that the observed dissociation behavior is not an artifact of individual metadynamics trajectories.

## 4 Dicsussion

ESCC is one of the most prevalent digestive malignancies, characterized by high morbidity and mortality. Patients are often diagnosed at advanced stages with lymph node metastasis, leading to poor prognosis and high mortality rates (Murphy et al., 2017). Previous studies have identified XIAP as a key regulator in various cancers, where its overexpression correlates with poor prognosis (Lou et al., 2021; Zohny et al., 2019; Łupicka-Słowik et al., 2020). While XIAP is primarily studied for its role in apoptosis inhibition, its impact on ESCC metastasis and patient outcomes remains unclear. Our previous research suggests that XIAP may promote tumor invasion and metastasis in ESCC and is associated with poor prognosis (Jin et al., 2019).

To further elucidate the clinical significance and potential mechanisms of XIAP in ESCC, we conducted systematic bioinformatics analyses to explore the expression characteristics and functional roles of XIAP in tumors. Pan-cancer analysis revealed that XIAP was significantly upregulated in 11 cancer types, including ESCC, suggesting its potential as a universal oncogenic factor. In ESCC, high XIAP expression was significantly correlated with shorter overall survival, and its expression level gradually increased with tumor stage progression, further supporting the critical role of XIAP in ESCC progression. Notably, single-cell sequencing analysis showed that XIAP was predominantly enriched in epithelial and endothelial cell clusters, which aligns well with our previous finding that XIAP promotes tumor migration by inducing epithelial–mesenchymal transition (EMT). Functional enrichment analysis further revealed that differentially expressed genes associated with high XIAP expression were mainly enriched in pathways related to extracellular matrix (ECM) remodeling, cell adhesion, and angiogenesis, including ECM–receptor interaction, focal adhesion, PI3K–Akt, TGF-β, and Notch signaling pathways. These pathways play central roles in tumor angiogenesis, invasion, and metastasis, suggesting that XIAP may promote the malignant progression of ESCC through regulation of these signaling pathways.

Collectively, these bioinformatics analyses not only validate the clinical significance of XIAP in ESCC but also provide new insights into the molecular mechanisms underlying its role in tumor progression. Given the critical role of XIAP in ESCC and its close association with angiogenesis, invasion, and metastasis-related pathways, therapeutic strategies targeting XIAP hold important potential for clinical translation.

In this study, we screened a series of natural products and structural derivatives, and identified a novel small-molecule compound, W-1-32, that can interact with XIAP. Spatial modeling and docking analysis demonstrated W-1-32’s stable binding to XIAP, with hydrogen bonding playing a crucial role in molecular recognition. To validate this interaction, we employed DARTS and CETSA assays, both confirming that W-1-32 directly binds to XIAP. Additionally, W-1-32 significantly inhibited ESCC cell proliferation and migration *in vitro*. Notably, Western blot analysis revealed a substantial reduction in XIAP protein expression following W-1-32 treatment. These findings suggest that W-1-32 may suppress ESCC invasion and migration by downregulating XIAP. Furthermore, XIAP gene silencing via shRNA attenuated the inhibitory effects of W-1-32 on ESCC proliferation and metastasis, consistent with XIAP-dependent activity of W-1-32 its effects through XIAP. However, we have to point that we have evaluated the effects of W-1-32 on ESCC invasion and metastasis only *in vitro*. To further validate its impact on ESCC metastasis, an *in vivo* ESCC lung metastasis model is still needed.

Although studies on ESCC-targeted therapy are ongoing, its overall efficacy remains suboptimal, and no first-line targeted therapy drugs are available in clinical practice. Small-molecule inhibitors derived from natural products have promising development potential, offering new avenues for identifying novel molecular-targeted drugs. Existing research indicates that embelin, a small-molecule XIAP inhibitor, exhibits antitumor activity across various malignancies by promoting the caspase pathway and inducing apoptosis through inhibition of XIAP binding to caspase family protein (Lee et al., 2017; Wang et al., 2020; Coyle et al., 2019). However, its lipophilicity and poor aqueous solubility have limited its extensive studies (Prabhu et al., 2018). SMAC mimetics are Smac/DIABLO-mimicking synthetic molecules that bind the BIR domains of XIAP and cIAP1/2 to induce cIAP degradation and drive TNF-dependent apoptosis. Representative agents such as birinapant and LCL161 have advanced to clinical trials but remain unapproved, underscoring significant challenges in clinical translation (Philchenkov and Miura, 2016). Accordingly, identifying potential XIAP inhibitors for ESCC therapies remains a critical research priority. The discovery of a novel XIAP inhibitor, W-1-32, provides a promising candidate for further research and ESCC therapeutic development.

W-1-32 is a natural product-derived XIAP modulator with a novel natural scaffold and favorable synthetic derivatization potential, thereby exhibiting unique properties distinct from currently available XIAP inhibitors. *In vitro* experiments demonstrated that W-1-32 exerted markedly lower cytotoxicity against normal esophageal epithelial cells than ESCC cells, indicating a potential tumor-selective profile that warrants further validation. As a structurally modifiable natural small-molecule derivative, W-1-32 possesses a tractable scaffold with favorable physicochemical amenability, which supports subsequent structural optimization and further medicinal chemistry development. However, compared with embelin and some SMAC mimetics that have entered clinical trials, W-1-32 still has several key limitations: its quantitative binding affinity for XIAP remains unclear, and no *in vivo* efficacy or toxicity data are available.

Post-translational modification plays a critical role in tumor invasion and metastasis (Johannessen et al., 2017). In this study, XIAP protein levels decreased following W-1-32 treatment in ESCC cells, while mRNA expression remained unchanged. Given that W-1-32 directly binds XIAP without altering its mRNA levels, we speculate that ligand engagement may promote XIAP destabilization potentially via the ubiquitin-proteasome pathway. However, this hypothesis remains unvalidated without direct proteasomal degradation (e.g., MG132 rescue) or ubiquitination assays. Elucidating whether degradation proceeds via ubiquitin-dependent proteasomal pathways, lysosomal pathways or direct conformational destabilization represents a key direction for future mechanistic studies. In summary, our findings suggest that W-1-32 has potential as a lead compound for XIAP-targeted agent development. This study provides a foundation for further development of W-1-32 and exploration of its anti-ESCC mechanism.

Computational analyses provided atomistic insight into the interaction between W-1-32 and XIAP. Electronic structure calculations (Figure 4b) revealed a balanced reactivity profile, with moderate chemical hardness and a polarized electrostatic surface, supporting stable yet adaptable noncovalent interactions. Molecular electrostatic potential mapping (Supplementary Figure S1) highlighted complementary electron-rich and electropositive regions that align with the physicochemical features of the XIAP binding pocket. Consistent with these features, docking and molecular dynamics simulations (Figure 7) indicated that W-1-32 adopts a stable binding pose within a defined XIAP cavity, maintained by coordinated hydrogen bonding and electrostatic contacts, as reflected by stable RMSD profiles, limited residue fluctuations, and confined conformational sampling. These findings are consistent with a binding mechanism dominated by noncovalent interactions rather than irreversible covalent modification, in agreement with the experimental observation of post-translational regulation of XIAP protein levels. Nevertheless, these simulations are inherently limited by force-field approximations and finite timescales, and therefore primarily provide a qualitative framework for understanding XIAP recognition by W-1-32.

Several limitations of the present study should be acknowledged. First, while target engagement of W-1-32 with XIAP was validated by CETSA and DARTS assays, quantitative binding affinity (e.g., K_d_ value) was not determined. Although XIAP knockdown attenuated the antitumor activity of W-1-32, neither rescue experiments nor direct detection of apoptotic signaling events was performed. Therefore, the current findings only support a strong correlation between XIAP binding. Second, all antitumor activity data presented herein are derived from *in vitro* assays. In the absence of *in vivo* efficacy and toxicity data, the *in vivo* pharmacological properties of the compound remain to be fully elucidated. Third, *in vitro* functional characterization of W-1-32 was limited to two differentially differentiated ESCC cell lines (Eca109, KYSE150). Despite consistent anti-proliferative and anti-migratory effects in both, further validation in expanded cell panels and patient-derived primary tumor cells is needed to assess generalizability across ESCC molecular heterogeneity.

## 5 Conclusion

This study identifies W-1-32 as a small-molecule modulator of XIAP in esophageal squamous cell carcinoma. Integrated electronic structure analysis, biophysical validation, and molecular simulations are consistent with direct XIAP engagement by W-1-32, accompanied by selective inhibition of ESCC cell proliferation, migration, and invasion. These findings establish a framework supported by converging computational, biophysical, and cell-based evidence for targeting XIAP-associated oncogenic processes. Future studies will be required to evaluate the *in vivo* pharmacological properties and translational potential of W-1-32, as well as to further elucidate the downstream signaling consequences of XIAP modulation in ESCC and related malignancies.

## Data Availability

The original contributions presented in the study are included in the article/Supplementary Material, and further inquiries can be directed to the corresponding authors.
