# Supplementary material for: A highly potent compound W-1-32 targeting XIAP protein inhibits proliferation and metastasis of esophageal squamous cell carcinoma
Source: Front Pharmacol. 2026 Aug 19;17:1902572. doi: 10.3389/fphar.2026.1902572 (PMC13533955; doi:10.3389/fphar.2026.1902572)
Supplement: Supplementary file 1 [file Supplementaryfile1.docx]

Supplementary Table S1. Total energy, frontier molecular orbital energies, and conceptual DFT descriptors of W-1-32.

| Total Energy | -2608.242272733407 Ha |  |
| --- | --- | --- |
| HOMO | -0.19588786 Ha | -5.3304 eV |
| LUMO | -0.07777737 Ha | -2.1164 eV |
| Gap (LUMO-HOMO) | 0.11811049 Ha | 3.2140 eV |
| Chemical hardness, η |  | 1.6070 eV |
| Chemical softness, S |  | 0.3110 eV^−1^ |
| Electronegativity, χ |  | 3.7234 eV |
| Chemical potential, μ |  | −3.7234 eV |
| Electrophilicity index, ω |  | 4.314 eV |

**Note**: Hartree, Ha.

**Supplementary Fig. 1 | Molecular electrostatic potential (MEP) surface of W-1-32.** The molecular electrostatic potential was mapped onto the electron density surface of W-1-32. Negative electrostatic potential regions (green) and positive potential regions (yellow) are shown. The global minimum potential (**Vmin = −0.097 a.u.**) is localized near the phenolic oxygen atom O26, indicating a dominant electron-rich site. The global maximum potential (**Vmax = 183.76 a.u.**) is located adjacent to atom C58 near the piperazine moiety, reflecting a strong electropositive region.
